# Low Interferon-γ Levels in Cord and Peripheral Blood of Pregnant Women Infected with SARS-CoV-2

**DOI:** 10.3390/microorganisms11010223

**Published:** 2023-01-16

**Authors:** Michele Cennamo, Evelina La Civita, Laura Sarno, Gianluigi Carbone, Sarah Di Somma, Serena Cabaro, Jacopo Troisi, Angelo Sirico, Francesco Paolo Improda, Maurizio Guida, Daniela Terracciano, Giuseppe Portella

**Affiliations:** 1 Department of Translational Medical Science, University of Naples “Federico II”, 80138 Naples, Italy; 2 Department of Neurosciences, Reproductive Science and Dentistry, University of Naples “Federico II”, 80131 Naples, Italy; 3 Department of Medicine, Surgery and Dentistry, “Scuola Medica Salernitana”, University of Salerno, 84081 Baronissi, Italy; 4 Ospedale San Pio-PO G Rummo, 82100 Benevento, Italy

**Keywords:** SARS-CoV-2, pregnancy, Interferon-γ, anti-Interferon antibody, immune system, cord blood

## Abstract

COVID-19 is characterized by the immune system’s overreaction resulting in a ‘cytokine storm’, consisting in a massive release of cytokine into the bloodstream, leading to local and systemic inflammatory response. This clinical picture is further complicated in case of infection of patients with a peculiar immunological status, such as pregnancy. In this paper, we focused on Interferon-γ, which plays a pivotal immunomodulatory role in normal pregnancy and fetal development, as well as in defense against pathogens. In this study, we compared the levels of Interferon-γ and the Interferon autoantibodies of the peripheral and cord blood of pregnant women with confirmed mild COVID-19 and healthy pregnant women. The Interferon-γ was significantly lower both in the peripheral and cord blood of SARS-CoV-2-positive mothers, suggesting that infection can affect the fetal microenvironment even without severe maternal symptoms. In conclusion, further studies are needed to clarify whether lower levels of Interferon-γ due to SARS-CoV-2 infection affect the development or infection susceptibility of infants born to SARS-CoV-2-infected mothers.

## 1. Introduction

Since its identification in China in late 2019, the SARS-CoV-2 epidemic has spread rapidly worldwide, affecting millions of people, pushing the World Health Organization (WHO) to declare the COVID-19 outbreak a global pandemic. The real extent of this health emergency is difficult to assess due to the swift increase in reported cases and the high number of undiagnosed asymptomatic cases (from 18% to 33%) [1,2], making it difficult to find an effective strategy to limit the SARS-CoV-2 spread [3,4,5].

The clinical spectrum of COVID-19 ranges from asymptomatic or paucisymptomatic forms to full-blown clinical presentations, characterized by acute respiratory failure requiring mechanical ventilation, septic shock and multiple organ failure. When present, symptoms are caused by an immune system’s overactivation, resulting in a ‘cytokine storm’ characterized by the release of high levels of cytokines, especially IL-6 and TNF-α, into the circulation, leading to a local and a systemic inflammatory response [6,7]. The onset in the majority of symptomatic patients is commonly characterized by fever, cough and shortness of breath and less commonly by sore throat, anosmia, dysgeusia, anorexia, nausea, myalgia and diarrhea.

Regarding the laboratory abnormalities, both leukopenia and leukocytosis, lymphopenia, thrombocytopenia, abnormal renal function parameters and elevated values of C-reactive protein levels (CRP), lactate dehydrogenase (LDH), D-dimer, erythrocyte sedimentation rate and procalcitonin have been reported [8,9,10].

This clinical picture becomes even more complicated in patients with a peculiar immunological status such as pregnancy, characterized by multiple developmental stages, each with unique immunological requirements, with the aim of protecting the fetus by preventing its identification as a non-self. Interferons (IFNs) play a pivotal immunomodulatory role in normal pregnancy and development, as well as in defense against pathogens [11,12], so it is possible to suppose that COVID-19 could affect the pregnancy course even in the case of asymptomatic infection. In addition, symptomatic pregnant mothers respond to infections differently, due to the combined effects of an altered general immune response and the peculiarity of a fetal–placental immune system, leading to an unpredictable disease [13,14]. Moreover, the respiratory changes that occur physiologically during pregnancy increase the susceptibility to severe COVID-19, per se [15].

To assess whether SARS-CoV-2 infection affects the maternal and fetal IFN secretion, we compared IFNγ levels in maternal blood and in cord blood in a pregnant population affected by COVID-19.

## 2. Materials and Methods

### 2.1. Study Population

Women and their newborns admitted to University Hospital Federico II between February and June 2021 were included in this study. Cases were pregnant women with SARS-CoV-2 infection hospitalized in the COVID-19 Unit of the Mother and Child Department and controls were pregnant women with uneventful pregnancies admitted to the Mother and Child Department. This population was mostly asymptomatic.

Exclusion criteria were maternal age < 18, unvaccinated patients, patients admitted in active labor, multiple gestation and premature rupture of membranes.

All patients were informed of the purpose and procedures of the study and signed a written consent form.

Clinical and anamnestic data were collected at admission and reported on a dedicated dataset. The study was conducted according to the ethical guidelines of the Helsinki Declaration of the World Medical Association and was approved by the ethics committee (protocol 140/20/ES2COVID19) of the University of Naples Federico II.

### 2.2. Sample Collection

Maternal whole blood samples and maternal serum samples were collected at admission for each patient using BD vacutainer (Becton Dickinson, Oxfordshire, UK) blood collection tubes containing EDTA and BD vacutainer with no additives, respectively; all patients were asked to respect a 12 h fast before blood collection. The whole blood sample was immediately analyzed for white cell count, while serum sample was separated from blood cells and immediately stored at −80 °C. Soon after the delivery and before the afterbirth, an arterial cord blood serum sample was collected and stored, as reported for maternal serum samples.

### 2.3. Biochemical Determination

White blood cell count was performed using the Siemens Advia 2120i hematology analyzer (Siemens Healthcare, Munich, Germany) according to the manufacturer’s recommendations. C-Reactive Protein was evaluated on Architect c16000 (C-Reactive Protein assay, Abbott Diagnostics, Chicago, IL, USA) according to the manufacturer’s recommendations.

SARS-CoV-2 infection was assessed by molecular analysis (RT-PCR) of the nasopharyngeal swab [16]. In detail, swabs were evaluated by Alinity m SARS-CoV-2 assay (Abbott Molecular Inc., Des Plaines, IL, USA), a real-time reverse-transcriptase polymerase chain reaction (rRT-PCR) test performed on the fully automated Alinity m system. The assay detects SARS-CoV-2 RNA from a volume of 500 µL. The Alinity m system automatically performs sample preparation, RT-PCR assembly, amplification, detection, results calculation and reporting. The Alinity m SARS-CoV-2 assay is a dual target assay (RNA-dependent RNA polymerase (RdRp) and nucleocapsid (N) genes) to detect the presence of the SARS-CoV-2 RNA. A sequence unrelated to SARS-CoV-2—from the pumpkin plant Cucurbita pepo—serves as a process control.

### 2.4. Determination of Interferon-γ (IFN-γ) and Anti-IFN-γ Antibodies

Sera were analyzed for IFN-γ and anti-IFN-γ antibodies performing two different ELISA assays; Human IFN-γ Uncoated ELISA quantitative detection of human IFN-γ INVITROGEN (Thermo Fisher Scientific, Eugene, OR, USA) and Human Anti-Interferon antibody ELISA Kit; (MyBioSource San Diego, CA, USA) in accordance with the manufacturers’ instructions.

### 2.5. Statistical Analysis

Data were analyzed with the GraphPad Prism 5 software package. Data were tested for normality using the D’Agostino–Pearson normality test. For not normally distributed data we used nonparametric tests. Statistical analysis was performed by an unpaired two-tailed *t*-test or two-tailed Mann–Whitney test, as indicated in figure legends.

Simple linear regression analysis of the data to assess the relationship between maternal and fetal IFN-γ and between maternal IFN-γ and lymphocytes were considered statistically significant when *p*-value was either equal to or less than 0.05.

## 3. Results

### 3.1. Characteristics of the Study Population

This study involved 25 mothers with COVID-19 and 24 controls. The main clinical and demographic characteristics of the study population are described in Table 1. There was no significant difference in terms of maternal age, comorbidity and parity.

### 3.2. Characteristics of the Newborns

All the included newborns were appropriate for their gestational age and they did not develop neonatal complications. Furthermore, there was no significant difference in neonatal length, weight and head circumference between the two groups (Table 2).

### 3.3. Effect of SARS-CoV-2 Infection on Blood Cell Count and CRP in Pregnant Women

**Figure 1:**
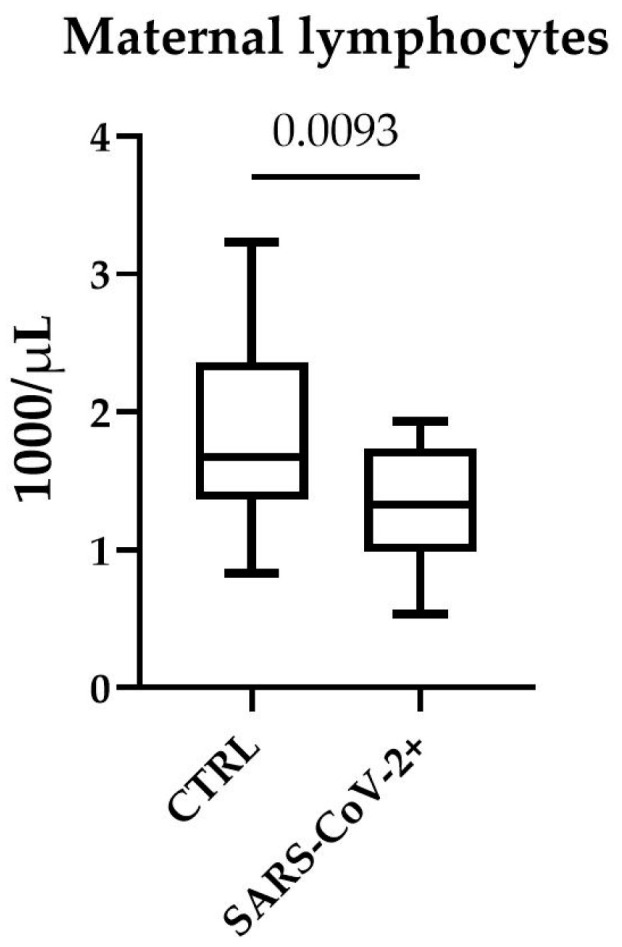
Lymphocyte count in maternal blood. Lymphocytes were counted in EDTA blood using ADVIA 2 analyzer. Lymphocyte concentration is expressed as 1000/µL. Box plots denote median and 25th to 75th percentiles (boxes) and min-to-max whiskers. The *p* value was evaluated using unpaired *t*-test.

We analyzed white blood cell counts and we measured the levels of CRP. In our study population, we did not find any significant difference between white blood cell count, neutrophils, monocytes and CRP (Table 3) in SARS-CoV-2-positive mothers compared to controls. Conversely, the lymphocyte count was significantly lower in pregnant women affected by COVID-19 (Figure 1) when compared to controls (*p* value 0.0093).

### 3.4. Measurement of IFN-γ and Anti IFN-γ Antibodies in Serum and Cord Blood of Mothers Affected by COVID-19

**Figure 2:**
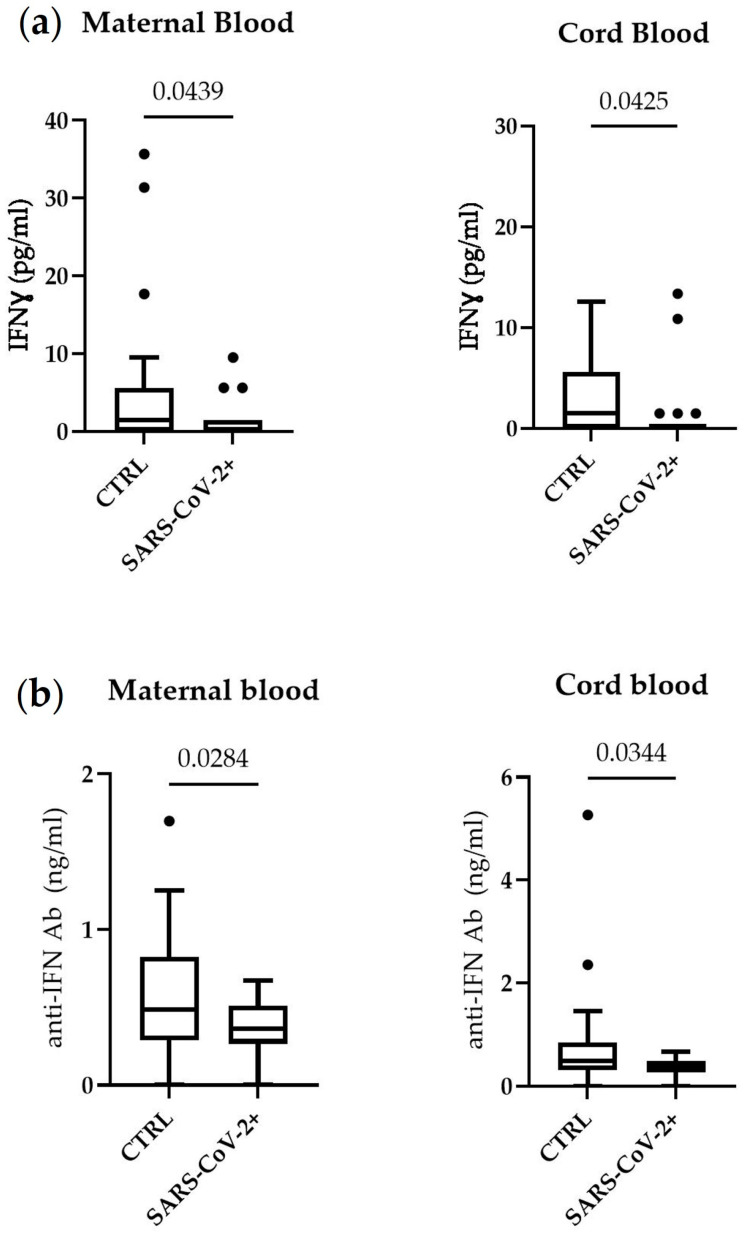
Serum IFN-γ in maternal blood and cord blood (**a**) and serum IFN antibodies (**b**). Box plots denote median and 25th to 75th percentiles (boxes) and Tukey whiskers. The *p* value was evaluated using unpaired *t*-test and Mann–Whitney test.

To determine if the decrease in lymphocytes is mirrored by lower levels of serum type II Interferon, we measured IFN-γ levels in the maternal and cord blood of infected mothers. As shown in Figure 2a, IFN-γ levels were significantly lower in both the maternal and cord blood of COVID19-positive mothers, suggesting that the maternal decrease in lymphocyte number affects maternal IFN-γ levels, which might lead to lower IFN-γ levels in cord blood.

In addition, to evaluate the potential involvement of an autoimmune mechanism explaining lower IFN-γ levels observed in SARS-CoV-2-positive mothers, we measured the anti-Interferon autoantibody levels in the maternal and cord blood (Figure 2b). In both the maternal and cord blood, the anti-Interferon antibodies were significantly lower in SARS-CoV-2-positive mothers compared to controls, indicating that autoantibodies are not involved in the IFN-γ decrease.

### 3.5. Correlation between IFN-γ Cord Blood and Maternal Blood

**Figure 3:**
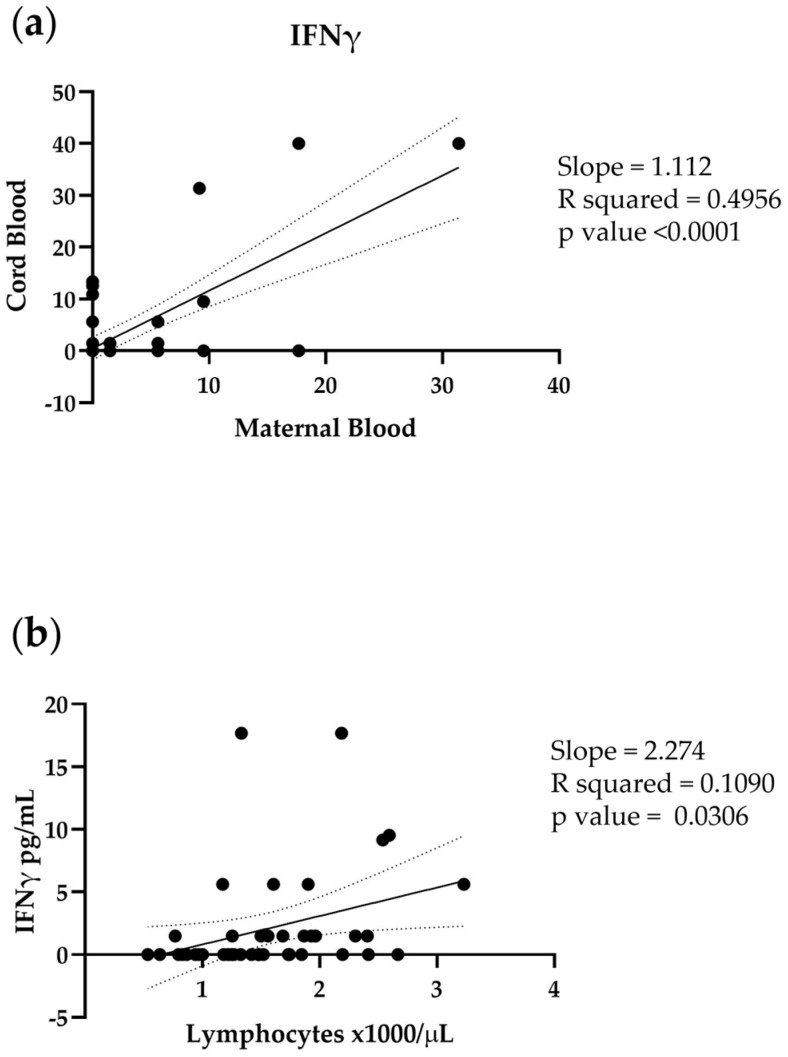
Simple linear regression of (**a**) IFN-γ levels (pg/mL) in maternal blood and cord blood; (**b**) lymphocyte count (1000/μL) and IFN-γ levels (pg/mL) in maternal blood. Each subject is represented on a scatter plot.

To assess whether the IFN-γ levels in cord blood reflect the maternal IFN-γ levels, we performed a simple linear regression, as shown in Figure 3a. The IFN-γ displayed a significantly positive correlation (*p* value < 0.0001), suggesting that blood IFN-γ levels mirrored the maternal values.

### 3.6. Correlation between Maternal IFN-γ and Maternal Lymphocyte Count

To further confirm that the lower levels of IFN-γ are caused by the depletion of maternal lymphocytes, we carried out a simple linear regression between maternal IFN-γ and lymphocyte count. IFN-γ positively correlated with the number of lymphocytes, suggesting that the lymphocyte reduction following the COVID-19 infection could lead to a lower level of maternal IFN-γ and, consequently, lower levels of cord blood IFN-γ.

## 4. Discussion

**Figure 4:**
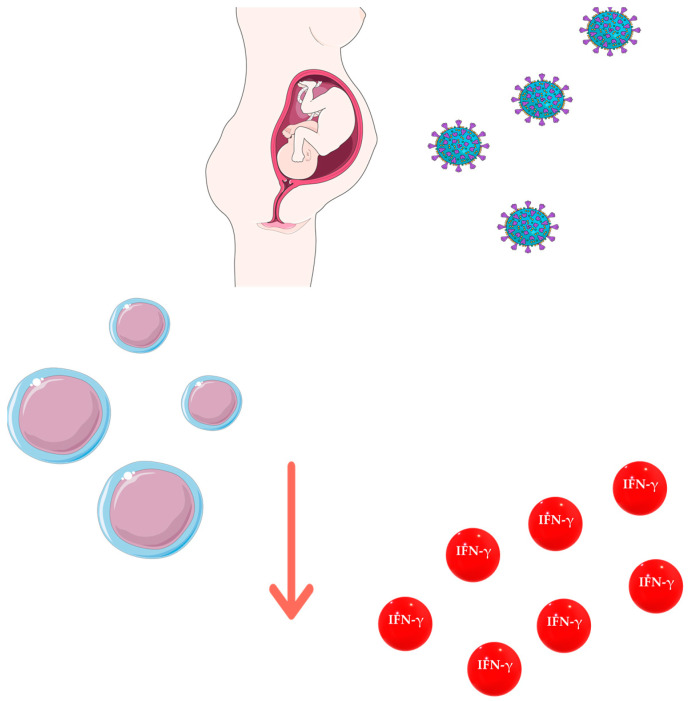
Schematic representation of lymphocyte and IFN-γ decrease in maternal and cord blood. Parts of the figure were drawn using pictures from Servier Medical Art by Servier, licensed under a Creative Commons Attribution 3.0 Unported License.

In our cohort, study we showed that IFN-γ is, significantly, decreased in COVID-19 pregnant women compared to controls in both maternal and cord blood samples (Figure 4).

COVID-19 was described as a trigger that leads to the fast activation of innate immune cells, with a profound cytokine response, resembling a hyper-inflammatory state. Tanacan et al., in fact, described pregnant women with COVID-19 as people with an alert immune system in which neutrophils, monocytes and lymphocytes were increased, as well as CRP [17]. In addition, the progression of the severe form does not seem to be exclusively related to viral load, but it may include defective Interferon responses [18]. A dysregulated inflammatory response to SARS-CoV-2 represents the main cause of disease severity and death in COVID-19 patients [19]. It is characterized by acute lymphopenia, elevated levels of circulating cytokines and substantial mononuclear cell infiltration in the lung, spleen, kidney, lymph nodes and hearth [20]. In patients affected by SARS-CoV-2, the proinflammatory response and the cytokine storm play a pivotal role in clinically relevant consequences for the host. When the immune system is not as able to counteract the virus and to conclude the inflammatory response, the aberrant production of the cytokines leads to macrophage hyperactivity, leading to fever, anemia and organ malfunction. At some point, the cytokine storm becomes unstoppable, leading to irreversible end-organ dysfunction and even death [21,22]. These studies examined patients with a severe disease, whereas our results are more likely related to the large portion of asymptomatic cases or those with mild symptoms. Other studies conjecture, in fact, that the identification of specific cytokines as indicators of disease severity might improve clinical management of COVID-19 patients, having a great impact on diagnostic and therapeutic decision making [23].

According to the literature, lymphocyte count decreased in our study population [24,25]. However, the mechanism leading to lymphopenia during COVID-19 still needs to be elucidated. It may be due to the direct contribution of the virus or redistribution of WBC via chemotaxis or apoptosis [26,27,28]. Nevertheless, the reduction of IFN-γ is likely due to lymphopenia both in maternal and, subsequently, cord blood.

Physiologically, Interferon (IFN) signaling plays an important role in restricting congenital infections, as well as in the physiology of healthy pregnancies. IFNs are best known for their antiviral activities: in mouse models, in fact, IFN signaling has an antiviral effect at the maternal–fetal interface and transplacental transmission [29,30,31]. In addition, IFN-γ inhibits the secretion of granulocyte/macrophage colony-stimulating factor, which promotes trophoblast growth and differentiation during normal pregnancy [32]. IFN-γ also inhibits trophoblast cell outgrowth, and trophoblast cell invasion is accelerated in mice with a genetic deficiency in the IFN-γ or the IFN-γ receptor [33].

Our results showed that serum IFN-γ auto-Ab concentration (including both neutralizing and non-neutralizing antibodies) is decreased both in maternal serum and cord blood. Conversely, we expected an inverse proportionality between IFN-γ and IFN-γ auto-Ab concentration, as previously described [34,35]. This unexpected finding could be explained with two possible hypotheses or with their combination. The first hypothesis, the most likely, could be that IFN-γ auto-Ab was not produced due to the lack of the antigenic stimulus (low concentration of IFN-γ) [36]. The second one could be related to the immunological status of pregnant women [37,38]. Whatever the reason, the low concentration of both IFN-γ and IFN-γ auto-Ab could be relevant to assess the clinical outcome. It has already been described, in fact, that the association of the low concentration of IFN-γ, the presence of neutralizing IFN-γ auto-Ab and the increased susceptibility to non-tuberculous mycobacteria disseminated infection in vulnerable hosts [39,40,41]. Thus, further studies are required to clarify whether children born to SARS-CoV-2-infected women will have worse outcomes than the general population.

Regarding neonatal outcomes, regarding neonatal length, birthweight, head circumference and Apgar score, we observed no significant differences between cases and controls. These findings are consistent with the literature, particularly in reference to the alpha variant of SARS-CoV-2 [42,43,44]. There are differences in the response of the immune system (in the mother and fetus) and symptoms among SARS-CoV-2 variants [45,46]. Nowadays, the most prevalent strain is omicron, but in the first semester of 2021, the most widespread variant was the alpha one; the B.1.1.7 lineage [47,48,49]. Thus, in our study population, the most represented variant was the alpha one. Further studies are needed to explore neonatal outcomes for other SARS-CoV-2 variants.

Our study has some limitations. Firstly, we were not able to investigate the outcomes of children born from women infected with SARS-CoV-2. Secondly, further studies are needed to clarify the molecular mechanism involved in the decrease in IFN-γ and IFN-γ auto-Ab.

## 5. Conclusions

In conclusion, our findings showed that Interferon-γ was significantly lower both in the peripheral and cord blood of SARS-CoV-2-positive mothers. Thus, infection can affect the fetal microenvironment even without severe maternal symptoms, suggesting the need for long-term follow-up of newborns born from SARS-CoV-2-infected pregnant mothers.

## Figures and Tables

**Table 1:** Characteristics of Study Population.

	SAR-CoV-2- Negative	SARS-CoV-2- Positive	*p* Value
Age (years)	33.5 ± 5.4	31.1 ± 4.2	0.0881
BMI (kg/m^2^)	29.3 [25.7; 32.9]	26.6 [22.5; 30.7]	0.1896
Comorbidity	4 (16%)	1 (4.2%)	0.3487
Parity			0.0865
One	5 (20%)	12 (50%)	
Two	11 (44%)	7 (29.2%)	
Three or more	9 (36%)	5 (20.8%)	
COVID-19 symptoms			
Presence of symptoms			
No		17 (70.8%)	
Yes		7 (29.2%)	
Fever		1 (14.3%)	
Mild respiratory symptoms		6 (85.7%)	
Anosmia		2 (28.6%)	
Gastro-intestinal symptoms		2 (28.6%)	
Dyspnea		0	
Pregnancy associated complications			
Gestational hypertension	0	1 (4.16%)	0.4898
Preeclampsia	0	0	
Gestational diabetes mellitus	0	0	
Neonatal outcomes			
SARS-CoV-2 infection	0	0	
Prematurity	1 (4%)	1 (4.16%)	
Apgar score			0.0828
≥9	16 (64%)	8 (33.3%)	
8	7 (28%)	14 (58.3%)	
≤7	2 (8%)	2 (8.4%)	
Data are expressed as median and range [25% percentile; 75% percentile] or as the number of cases (%). Age is expressed as media ± SD. BMI: body mass index.			

**Table 2:** Characteristics of the newborns. Anthropometric characteristics of newborns are expressed as mean (± SD).

Characteristic	Children Born to SARS-CoV-2-Negative Mothers	Children Born to SARS-CoV-2-Positive Mothers	*p* Value
Weight	3.2 ± 0.489 Kg	3.1 ± 0.536 Kg	0.5879
Length	49.53 ± 1.48 cm	49 ± 1.82 cm	0.5384
Head Circumferences	34.18 ± 1.31 cm	35 ± 1.08 cm	0.2525

**Table 3:** Laboratory parameters in maternal blood.

	SARS-CoV-2- Negative	SARS-CoV-2- Positive	*p* Value
PCR (mg/L)	5.5 (2.6; 7)	4.8 (2.3; 13.7)	0.76 ^b^
WBC (×1000/μL)	10 (7.3; 12.42)	9.5 (6.63; 12.38)	0.78 ^a^
Neutrophils (×1000/μL)	7.05 (5.30; 9.23)	6.92 (4.7; 9.1)	0.87 ^a^
Lymphocytes (×1000/μL)	1.67 (1.48; 2.31)	1.3 (1; 1.7)	0.0093 ^a^
Monocytes (×1000/μL)	0.54 (0.34; 0.72)	0.51 (0.34; 0.62)	0.42 ^a^
Platelets (×1000/μL)	201 (165.7; 246.75)	198.5 (179.2; 251.5)	0.66 ^a^
Results are expressed as median and range [25% percentile; 75% percentile]— ^a^: unpaired *t*-test; ^b^: Mann–Whitney U test			

## Data Availability

The data presented in this study are available on request from the corresponding author. The data are not publicly available in accordance with the guidelines of the Ethics Committee.
